# P-1358. Efficacy of combination therapy with minocycline for the treatment of Stenotrophomonas maltophilia infections

**DOI:** 10.1093/ofid/ofaf695.1545

**Published:** 2026-01-11

**Authors:** Tuvanont Pongdumbun, Yong Rongrungruang, Adhiratha Boonyasiri

**Affiliations:** Division of Infectious Diseases, Department of Internal Medicine, Buddhachinaraj Hospital, Bangkok, Krung Thep, Thailand; Division of Infectious Diseases and Tropical Medicine, Department of Medicine, Faculty of Medicine Siriraj Hospital, Mahidol University, Bangkok, Thailand., Bangkok, Krung Thep, Thailand; Department of Research and Development, Faculty of Medicine Siriraj Hospital, Mahidol University, Bangkok, Thailand, Bangkok, Krung Thep, Thailand

## Abstract

**Background:**

*Stenotrophomonas maltophilia* is a multidrug-resistant bacterium that causes high in-hospital mortality worldwide. However, published data on the utility of combination therapy for *S. maltophilia* infections remain limited.

**Methods:**

We enrolled adult patients with *S. maltophilia* infection in a randomized, double-blind, placebo-controlled, single-center clinical trial at Siriraj Hospital, Thailand. Patients were assigned 1:1 to receive minocycline capsules (100 mg twice daily) in combination with either levofloxacin or trimethoprim-sulfamethoxazole or monotherapy with either levofloxacin or trimethoprim-sulfamethoxazole. The study outcomes were 28-day mortality, clinical and microbiological responses, and adverse events. The favorable microbiological outcome was that the previously identified pathogen was not detected, and no new pathogen was detected.

**Results:**

Between 2022 and 2024, 112 patients were randomly assigned to combination therapy (n = 55) or monotherapy (n = 57). Most patients were in the intensive care unit at the time of enrollment (71.4%). There was no difference in 28-day mortality between groups (43.6% combination therapy vs 38.6% monotherapy, *p* = 0.59). Patients on combination therapy had significantly more favorable microbiological outcomes than patients on monotherapy 72 hours after treatment (45.5% on combination therapy vs 14% on monotherapy, *p* < 0.001) and at the end of treatment (80% on combination therapy vs 56.1% on monotherapy, *p* = 0.023). However, we observed comparable favorable clinical response rates 72 hours after treatment and at the end of treatment (52.7% on combination therapy vs 42.1% on monotherapy, *p* = 0.30, and 54% on combination therapy vs 57% on monotherapy, *p* = 0.86, respectively). Acute kidney injury was independently associated with 28-day mortality per a multivariate analysis. Adverse events, including acute kidney injury (n = 65/112; 58%) and elevated liver enzymes (n = 8/112; 7%), occurred but were not related to the study drug.

**Conclusion:**

Combination therapy with minocycline had better microbiological efficacy than monotherapy in patients with *S. maltophilia* infection and was safe. However, a beneficial effect of combination therapy with minocycline on the clinical outcomes of *S. maltophilia* infections was not noted.

**Disclosures:**

All Authors: No reported disclosures

